# Functional deficits of the attentional networks in autism

**DOI:** 10.1002/brb3.90

**Published:** 2012-08-27

**Authors:** Jin Fan, Silvia Bernardi, Nicholas T Dam, Evdokia Anagnostou, Xiaosi Gu, Laura Martin, Yunsoo Park, Xun Liu, Alexander Kolevzon, Latha Soorya, David Grodberg, Eric Hollander, Patrick R Hof

**Affiliations:** 1 Department of Psychology, Queens College, City University of New YorkNew York; 2 Department of Psychiatry, Mount Sinai School of MedicineNew York; 3 Fishberg Department of Neuroscience and Friedman Brain Institute, Mount Sinai School of MedicineNew York; 4 Seaver Autism Center for Research and Treatment, Mount Sinai School of MedicineNew York; 5 Albert Einstein College of Medicine and Montefiore Medical CenterNew York

**Keywords:** Alerting, anterior cingulate cortex, attentional networks, autism, executive control

## Abstract

Attentional dysfunction is among the most consistent observations of autism spectrum disorders (ASD). However, the neural nature of this deficit in ASD is still unclear. In this study, we aimed to identify the neurobehavioral correlates of attentional dysfunction in ASD. We used the Attention Network Test-Revised and functional magnetic resonance imaging to examine alerting, orienting, and executive control functions, as well as the neural substrates underlying these attentional functions in unmedicated, high-functioning adults with ASD (*n* = 12) and matched healthy controls (HC, *n* = 12). Compared with HC, individuals with ASD showed increased error rates in alerting and executive control, accompanied by lower activity in the mid-frontal gyrus and the caudate nucleus for alerting, and by the absence of significant functional activation in the anterior cingulate cortex (ACC) for executive control. In addition, greater behavioral deficiency in executive control in ASD was correlated with less functional activation of the ACC. These findings of behavioral and neural abnormalities in alerting and executive control of attention in ASD may suggest core attentional deficits, which require further investigation.

## Introduction

Autism spectrum disorders (ASD) are neurodevelopmental disorders characterized by deficits in social interaction, communication, as well as stereotyped and repetitive behaviors, and restricted interest in domains of activity. Although attentional dysfunction is one of the most consistently reported cognitive deficits in autism (Allen and Courchesne, 2001), the specific components and component interactions in the attentional networks that are impaired in ASD remain unclear. An investigation of attentional functions and related brain networks could provide more comprehensive information about potentially important core deficits for research, diagnosis, and treatment of ASD.

We conceptualize attention as consisting of three distinct functional components: alerting, orienting, and executive control (Posner and Fan 2008). The *alerting* function subsumes the capacity to increase vigilance tonically (i.e., increased vigilance related to increased general arousal), or phasically (i.e., increased vigilance related to a specific stimulus) to process an impending stimulus. The *orienting* function supports the selection of specific information from numerous sensory inputs. Orienting involves rapid or slow shifting of attention among targets within or between modalities, with three elementary operations: disengaging attention from its current focus, moving attention to the new target, and engaging attention at the new target (Posner et al. 1984). The *executive control* of attention involves the engagement of more complex mental operations during detection and resolution of conflict between competing goals or functions.

Each of the three attentional functions is mediated by anatomically distinct neural networks (Fan et al. 2005). Alerting has been associated with the thalamus and the temporoparietal junction (TPJ) and other parietal regions (Fan et al. 2005). Additionally, the mid-frontal gyrus (MFG – a part of the dorsolateral prefrontal cortex, DLPFC), as well as the caudate nucleus and putamen, has been implicated in efficient processing of warning signals involved in generating an anticipatory response (Fan et al. 2007; Clerkin et al. 2009). The orienting system for visual events has been associated with the superior parietal lobule and the frontal eye fields (FEF) (Corbetta and Shulman 2002). It has been shown that the areas near and along the intraparietal sulcus (IPS) bilaterally and the FEF are involved in orienting, whereas the right TPJ and inferior frontal gyrus are involved in reorienting (Corbetta et al. 2008). Finally, executive control of attention involves the anterior cingulate cortex (ACC) and DLPFC (Matsumoto and Tanaka 2004). A number of neuroimaging studies have shown activation of the dorsal ACC in tasks requiring subjects to respond to one dimension of a stimulus instead of another strong, conflicting dimension (e.g., Bush et al. 2000; Botvinick et al. 2001; Fan et al. 2003).

Individuals with ASD have shown deficits in all three attentional functions. The Continuous Performance Test (CPT) (Rosvold et al. 1956) is the most commonly used paradigm for exploring the alerting function in autism; most results suggest a normal ability of ASD individuals to sustain attention (Garretson et al. 1990; Siegel et al. 1992; Pascualvaca et al. 1998). However, when the AX version of the CPT (subject responds to the target “X” when it is preceded by an “A” compared with the target preceded by other letters) was employed, children with autism showed a trend of benefiting less from the “A” cue, suggesting an abnormal phasic alerting function (Pascualvaca et al. 1998).

Orienting deficits are shown in tasks that require rapid shifting of attention between modalities (Courchesne et al. 1994a), between object features (Courchesne et al. 1994a,b; Rinehart et al. 2001), and between spatial locations (Wainwright-Sharp and Bryson 1993; Townsend et al. 1996a,b, 1999; Wainwright and Bryson 1996; Harris et al. 1999; Belmonte 2000). These deficits occur for auditory and visual targets separately (Lovaas et al. 1971, 1979; Townsend and Courchesne 1994) and jointly (Casey et al. 1993), as well as across different manipulations of attention adjusting and updating the scope of attention (Burack et al. 1997), engaging visual attention (Burack 1994), and disengaging attention (Wainwright and Bryson 1996). Orienting deficits in autism have been shown to be related to abnormalities in parietal lobe structure (Courchesne et al. 1993; Townsend and Courchesne 1994). Although many studies have shown that orienting deficits in individuals with autism are related to social cues (e.g., Dawson et al. 1998), especially human faces, other studies provide evidence of nonspecific orienting deficits (Landry and Bryson 2004; Teder-Salejarvi et al. 2005). Although deficits in spatial orienting have been documented (e.g., Casey et al. 1993; Townsend et al. 1996a) and have been shown to relate to structural abnormalities in the cerebellum and parietal lobe (Courchesne et al. 1993; Townsend et al. 1996a), the neural mechanisms of orienting deficits, especially in the context of joint attention, still remain unclear.

Behavioral studies have been conducted to examine whether there are deficits in executive control of attention in ASD using cognitive paradigms such as the Go/No-Go and the Stroop tasks. Although executive control dysfunction may be attributed to frontal lobe abnormalities that have been observed in individuals with autism (Courchesne et al. 2001; Sparks et al. 2002), there is no consistent evidence supporting impaired inhibition, for example, on the Stroop task (Russell et al. 1999) or the Go/No-Go task (Ozonoff and McEvoy 1994). One study, examining conflict processing, found no group differences in mean ACC activation during functional magnetic resonance imaging (fMRI); however, the results indicated an abnormal time course of the hemodynamic response in this region during conflict conditions (Dichter and Belger 2007). Evidence also suggests abnormal functional connectivity between ACC and other important regions in ASD (Welchew et al. 2005; Kana et al. 2007). Abnormal behavioral performance in conflict processing, significant metabolic reduction in the ACC (Haznedar et al. 1997), and abnormal ACC activation and connectivity together suggest a prominent role of the ACC in impaired executive control in ASD.

Recent results suggest that the three attentional networks communicate with and influence one another to support the functional integration and interaction of attention (Fan et al. 2009). The overfocused or selective attention found in individuals with autism (Lovaas et al. 1979) may reflect abnormal interactions among attentional networks and core deficits of executive control, rather than a narrowed spotlight of visuospatial attention. Most prior studies on this topic were conducted using separate tasks not designed to investigate interactions among attentional networks. Thus, interactions among attentional networks in individuals with ASD compared with healthy controls (HCs) would seem to be a particularly important area of examination.

We examined the functions and neural mechanisms of the three attentional networks in individuals with ASD using the Attention Network Test-Revised (ANT-R) (Fan et al. 2009), probing attentional functions and allowing analysis of the functional integration and interaction of the attentional networks. We hypothesized deficits in the alerting, orienting, and executive control networks, and abnormal interaction among these networks in the ASD group relative to HCs.

## Method

### Participants

All eligible participants underwent a diagnostic evaluation consisting of psychiatric, medical, and developmental assessment (see Table 1 for demographic and clinical data). Intelligence quotient (IQ) was measured using the Wechsler Adult Intelligence Scale, third edition (WAIS-III) (Wechsler 1997). Fourteen high-functioning adults with autistic disorder or Asperger's syndrome (ASD group) and 14 healthy control (HC group) participants were recruited for this study at the Seaver Autism Center for Research and Treatment, Mount Sinai School of Medicine (MSSM). HCs were matched with patients on average IQ (within 15 points, 1 SD), age (birth date within 24 months), gender, and handedness. Handedness scores were measured by administering the Edinburgh Handedness Inventory (Oldfield 1971). Participants with ASD were diagnosed with autism or Asperger's syndrome by psychiatric interview according to the *Diagnostic and Statistical Manual-IV* Text Revision (*DSM-IV-TR*). These diagnoses were confirmed by the Autism Diagnostic Interview-Revised (ADI-R; Lord et al. 1994) and Autism Diagnostic Observation Schedule-Generic (ADOS-G; Lord et al. 2000), except for one participant for whom ADI-R was unavailable.

**Table 1:** Demographic data (means ± SD) of ASD and HC groups

Participant characteristics	ASD (*n* = 12)	HC (*n* = 12)	*t*	*P*
Age (years)	30 ± 6	28 ± 7	0.85	0.41
Sex (male/female)	9M/3F	10M/2F	0.49^1^	0.62
Handedness score	69 ± 37	75 ± 47	0.34	0.74
Years of education	15.6 ± 2.2	15.8 ± 1.7	0.25	0.83
Full scale IQ	115 ± 14	120 ± 15	0.84	0.41
Verbal IQ	116 ± 17	120 ± 15	0.75	0.46
Performance IQ	112 ± 15	116 ± 11	0.65	0.52
ASD diagnosis (autism/Asperger)	8/4			
ADI-R^2^	38.4 ± 13.4			
Social	18.8 ± 8.0			
Verbal communication	12.9 ± 4.0			
Repetitive behavior	6.7 ± 3.6			
ADOS-G	12.2 ± 4.1			
Communication	3.0 ± 1.8			
Social	7.3 ± 2.5			
Imagination	0.8 ± 0.7			
Stereotyped behaviors	1.3 ± 1.3			

ASD, autism spectrum disorder; HC, healthy control; IQ, intelligence quotient; ADI-R, Autism Diagnostic Interview-Revised; ADOS-G, Autism Diagnostic Observation Schedule-Generic.

1 Mann–Whitney *U* test.

2 ADI-R scores were not available for one participant, therefore *n* = 11 for this measure.

Exclusion criteria included epilepsy, history of schizophrenia, schizoaffective disorder, or other Axis I mental disorders, except attention-deficit hyperactivity disorder or obsessive-compulsive disorder (given the phenotypic overlap with ASD), and use of depot neuroleptic medication or other psychoactive drugs within the past 5 weeks. We also excluded potential participants with a lifetime history of substance/alcohol dependence and or substance/alcohol abuse within the last year. Additional exclusion criteria included history of encephalitis, phenylketonuria, tuberous sclerosis, fragile X syndrome, anoxia during birth, neurofibromatosis, hypomelanosis of Ito, hypothyroidism, Duchenne muscular dystrophy, and maternal rubella. Potential HCs were excluded based on medical illness or history in first-degree relatives of developmental disorders, learning disabilities, autism, affective disorders, and anxiety disorders.

Two ASD participants and two HC participants were excluded from the final sample due to indications from a neuroradiologist report of abnormal brain structure, low (chance-level) accuracy, motion greater than one voxel size, or technical issues resulting in the absence of behavioral data, with one participant in each of these categories. The final sample for this report included 12 ASD (eight with autism and four with Asperger's syndrome) and 12 HC participants. All participants provided written informed consent, approved by the MSSM Institutional Review Board.

### The Attention Network Test – Revised

**Figure 1:**
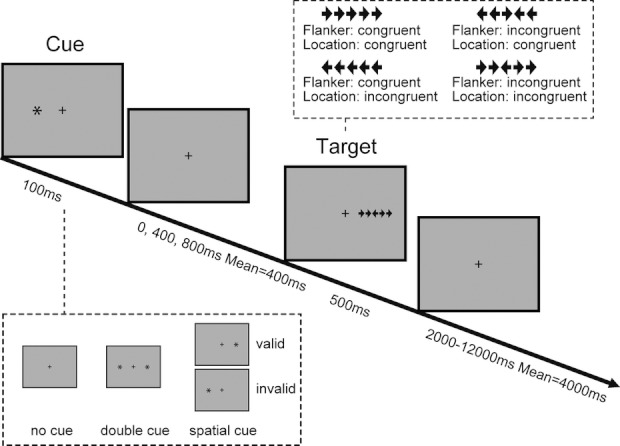
The schematic of the Attention Network Test-Revised (ANT-R). In each trial, depending on the cue condition (none, double, and valid or invalid cues), an asterisk “*” as the cue appears for 100 msec. After a variable duration (0, 400, or 800 msec), the target (the center arrow) and two flanker arrows on the left and right side (congruent or incongruent flankers) are presented for 500 msec. The participant makes a response to the target's direction. The post-target fixation period jitters between 2000 and 12,000 msec.

The ANT-R is a revision of the original ANT (Fan et al. 2002) aimed at optimizing attentional contrasts, as described in our previous publication (Fan et al. 2009). A minor difference between the task used in the current fMRI study and our previous behavioral study (Fan et al. 2009) is that asterisks, instead of flashing boxes, were used in the cue conditions (see Fig. 1). The participants' task was to respond to the direction that the center arrow (target) was pointing (either left or right) using the left index finger for the left direction and the right index finger for the right direction. The four flanker arrows, two on the left and two on the right side of the target, were either pointing to the same direction as the target (congruent condition) or the opposite direction (incongruent condition). The cue-to-target intervals (0, 400, and 800 msec) were selected based on previous studies with normal participants and patients with parietal damage (Posner et al. 1984; Fan et al. 2002). The ANT-R was compiled and run on a personal computer using E-Prime™ software (Psychology Software Tools, Pittsburgh, PA).

The function of each of the three attentional networks is operationally defined as a comparison of the performance (reaction time or accuracy) between one condition and the appropriate reference condition, resulting in scores for the attentional networks (Fan et al. 2009). For the alerting network, the phasic alerting (benefit) effect is defined as follows: Alerting = RT_no cue_ − RT_double cue_ representing the benefit of alerting. For the orienting network, the validity includes the ability to disengage attention from a previous location and to move and engage attention at a new location. Correspondingly, orienting operations are defined as follows: Validity = Disengaging + (Moving + Engaging)= RT_invalid cue_ − RT_valid cue_, which represents both the cost of an invalid cue and benefit of a valid cue. The validity effect has two subcomponents, disengaging and moving/engaging: (1) Disengaging = RT_invalid cue_ − RT_double cue_ for the cost of disengaging from invalid cue; (2) Moving + Engaging= RT_double cue_ − RT_valid cue_, for the benefit of target response under the valid cue condition. The Moving + Engaging is equivalent to the computation of “orienting” defined in our previous study (Fan et al. 2002). In addition, Orienting time = RT_valid cue, 0 msec cue-to-target interval_ −RT_valid cue, 800 msec cue-to-target interval_ is defined for the benefit of the target response because of the advanced orienting under the 800-msec cue-target interval condition. The conflict effect, which is a cost, is defined as follows: Flanker conflict = RT_flanker incongruent_ − RT_flanker congruent_. We have previously shown that the location incongruency effect (whether the location of the target – left or right – is on the same side as the target is pointing) is very small (Fan et al. 2009), and thus, we did not examine this effect- or location-related interactions in this study.

The interaction effects are defined as follows: (1) Alerting by flanker conflict = (RT_no cue, flanker incongruent_ − RT_no cue, flanker congruent_) − (RT_double cue, flanker incongruent_ − RT_double cue, flanker congruent_). A negative value indicates a negative impact of alerting on flanker conflict processing. (2) Orienting by flanker conflict = (RT_double cue, flanker incongruent_ −RT_double cue, flanker congruent_) − (RT_valid cue, flanker incongruent_ − RT_valid cue, flanker congruent_). A positive value indicates more efficient conflict processing because of valid orienting. (3) Validity by flanker conflict = (RT_invalid cue, flanker incongruent_ −RT_invalid cue, flanker congruent_) − (RT_valid cue, flanker incongruent_− RT_valid cue, flanker congruent_). A positive value indicates less efficient flanker conflict processing because of invalid orienting. The effects in error rate follow the same formulas.

### Event-related fMRI

Event-related fMRI was used to study the activation of the attentional networks. The time interval between the onset of the target and the next trial was jittered. The duration between the offset of the target and the onset of the next trial was varied systematically with a set of 12 discrete times from 2000 to 12,000 msec, including 10 intervals from 2000 to 4250 msec with an increase step of 250-, 4750-, and 12,000-msec intervals, approximating an exponential distribution with a mean of 4000 msec. The mean trial duration was 5000 msec. The response collection window was 1700 msec from onset of the target and the flankers. There were four runs in this experiment with 72 test trials in each. The total duration for each run was 420 sec. Total time required to complete this task was about 30 min.

### Data acquisition and analysis

Stimuli were presented at the center of the participant's field of view through a super video graphics array liquid crystal display projector system onto a rear-projection screen mounted at the back of the magnet bore. Participants viewed stimuli via a mirror attached to the head coil and positioned above their eyes. Participants responded with both hands using the BrainLogics fiber optic button system (Psychology Software Tools, Pittsburgh, PA).

Laboratory testing and training occurred outside of the scanner prior to the scan. In the scanner, participants viewed the stimuli and provided responses, recorded via computer, as measures of reaction time and accuracy. Mean RTs under the cue-by-target conditions were calculated after excluding the error trials. Error rates under each of these conditions were also calculated. Because behavioral data often have nonnormal distributions, skewness and kurtosis statistics were examined independently for each group for each variable. Any variable that exhibited both a skewness and kurtosis value greater than 1 was subject to nonparametric analysis, using the Mann–Whitney *U* statistic. All other between-group analyses were examined using parametric statistics.

#### Image acquisition

All MRI acquisitions were obtained on a 3 T Siemens Allegra MRI system at Mount Sinai School of Medicine. Each scanning run started with two dummy volumes before the onset of the task to allow for equilibration of T1 saturation effects, followed by 168 image volumes. All images were acquired along axial planes parallel to the anterior commissure–posterior commissure (AC–PC) line. A high-resolution T2-weighted anatomical volume of the whole brain was acquired on an axial plane parallel to the AC–PC line with a turbo spin-echo pulse sequence with the following parameters: 40 axial slices 4-mm thick, skip = 0 mm, repetition time (TR) = 4050 msec, echo time (TE) = 99 msec, flip angle = 170°, field of view (FOV) = 240 mm, matrix size = 448 × 512, voxel size = 0.47 × 0.47 × 4 mm. Four runs of T2*-weighted images were acquired with a gradient echo-planar imaging sequence using the following parameters: 40 axial slices 4-mm thick and skip = 0 mm, TR = 2500 msec, TE = 27 msec, flip angle = 82°, FOV = 240 mm, matrix size = 64 × 64.

#### Image analysis

Event-related analyses of the functional imaging data from the ANT-R sessions were conducted using statistical parametric mapping (SPM2; Wellcome Trust Centre for Neuroimaging, London, UK). The functional scans were realigned to the first volume, coregistered with the T2-weighted anatomical image, normalized to a standard template (MNI: Montreal Neurological Institute), resampled to 2 × 2 × 2 mm^3^, and spatially smoothed with an 8 × 8 × 8-mm full-width-at-half-maximum Gaussian kernel. Event-related analyses were performed using the default SPM basis function, which consists of a synthetic hemodynamic response function (HRF) composed of two gamma functions.

General linear modeling was conducted for the functional scans from each participant by modeling the measured event-related blood oxygen level–dependent (BOLD) signals and regressors to identify the relationship between the experimental events (i.e., the various manipulations in the ANT-R) and the hemodynamic response. Regressors were created by convolving a train of delta functions representing the sequence of individual events with the SPM basis function. The regressors included five cue-related HRFs: double cue, left valid cue, right valid cue, left invalid cue, right invalid cue; and 16 target-related HRFs: four cue conditions (no cue, double cue, valid cue, invalid cue) × two flanker conditions (congruent and incongruent) × two target locations (left and right). The six parameters generated during motion correction were entered as covariates. The specific effects of attentional processes were tested by applying linear contrasts to the regressors, such that for the conflict effect, the contrast of incongruent (eight regressors) minus congruent (eight regressors) conditions was used. The target responses under different cue-by-target conditions were equally weighted for the contrast between congruent and incongruent conditions. For fMRI analysis, the following attentional network effects were defined differently. For the alerting effect, the contrast was defined as double cue vs. baseline. Moving+ engaging was flipped as valid cue minus double cue. In addition, orienting was defined as spatial cue (valid+ invalid) minus double cue.

The images of contrast estimates from all participants were entered into a second-level group analysis conducted with a random-effect statistical model. An uncorrected *P*-value of 0.01 for the height (intensity) threshold of each activated voxel and an uncorrected *P*-value of 0.05 for extent threshold were simultaneously applied. This height and extent threshold combination is similar to the threshold suggested to reach a desirable balance between Type I and Type II errors (Lieberman and Cunningham 2009). The resultant statistical maps thresholded for height and extent protect against an inflation of the false-positive rate. Prior Monte Carlo simulations confirm the present voxel contiguity threshold (see Fan et al. 2011).

For the region-of-interest (ROI) analysis, we extracted the regression coefficients (β values) from the incongruent minus congruent contrast using a sphere with a 6-mm radius centered on the voxel of local maxima, identified based on group differences. The β values of ROIs are independent from the measures of RT and accuracy in the regression analyses. We examined between-group differences in the slope (which is independent of the main effect of group difference) of the regression models of the conflict effects (in error rate and RT) as a function of the brain activity related to conflict processing (contrast between incongruent and congruent conditions) at the group level. In this analysis, behavioral conflict effects were dependent variables, with ACC activation (extracted from the ROI peak = [−2, 34, 24]), group, and the interaction term of ACC activation-by-group variables as predictors. To explore whether the deficits in conflict processing are associated with clinical symptoms, we also conducted correlation analyses on the relationship between the measures of neuronal and behavioral effects, and the ADI-R and ADOS-G diagnostic algorithm total raw scores and subscale scores. An uncorrected *P*-value of 0.01 was used.

Due to preexisting group differences in error rates (and potential related ACC activation), error trials were modeled neither at the individual level nor as a covariate at the group level to avoid specification error, an inappropriate use of analysis of covariance to deal with substantive group differences on potential covariates (Miller and Chapman 2001). Given the large literature on cognitive deficits in ASD, increased conflict effect in error rate is not viewed as a covariate but rather as a feature of the disorder.

## Results

### Differences in behavioral performance

One sample *t*-tests with both groups combined showed that the attentional effects (in RT) of alerting, validity, disengaging, moving + engaging, orienting time, and flanker conflict were significant (*P* < 0.01). The validity by flanker was also significant (*P* < 0.05), although alerting by flanker effect was not significant (*P* > 0.05). For the error rate, the effects of alerting (*P* < 0.05), validity (*P* < 0.01), disengaging (*P* = 0.01), orienting time and flanker conflict (*P* < 0.01), and validity by flanker (*P* < 0.05) were significant, but moving + engaging, alerting by flanker, and orienting by flanker were not significant.

**Figure 2:**
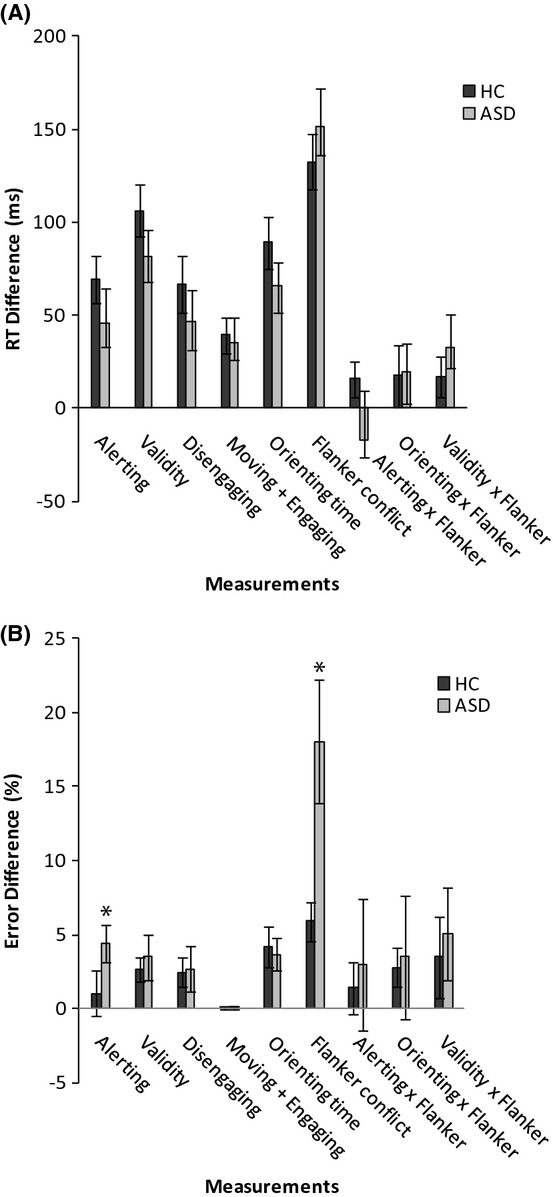
Behavioral performances measured by reaction time (RT) (A) and error rate (B) for each measurement for the groups of healthy controls (HC) and individuals with autism spectrum disorders (ASD). Error bars represent the standard error for each measurement. Note: *p < 0.05

Comparing the two groups, the mean overall accuracy for HC and ASD groups was 92 ± 6 and 79 ± 12% (mean and standard deviation), respectively; mean overall RTs for these two groups were 883 ± 161 and 878 ± 164 msec, respectively. The ASD group made significantly more errors than the HC group (13% difference), *t*_(22)_ = 3.26, *P* < 0.01, but the difference in overall RT (6 msec) was not significant, *t*_(22)_ = 0.09, *P* > 0.05. Figure 2 shows the network scores in RT and error rate, respectively. Although there were no significant group differences in RT, nonparametric statistical analyses showed a significant group difference in alerting-related errors, Mann–Whitney *U* = 34.5, *n*_1_ = *n*_2_ = 12, *P* < 0.05. The ASD group (*M* = 4.4%, *MDN* = 4.3%) made significantly more errors than the HC group (*M* = 1.0%, *MDN* = 0.0%) when the target appeared without, compared with, an alerting cue. The conflict effects for HC and ASD in error rate were 6 ± 4 and 18 ± 15% (greater variance in ASD), respectively, and in RT were 132 ± 52 and 151 ± 72 msec, respectively. The ASD group made significantly more errors than the HC group (18.1 vs. 5.9%) under the incongruent compared with the congruent target condition, *t*_(13.03)_ = 2.76, *P* < 0.05.

### Differences in functional activation associated with the attentional processes

**Figure 3:**
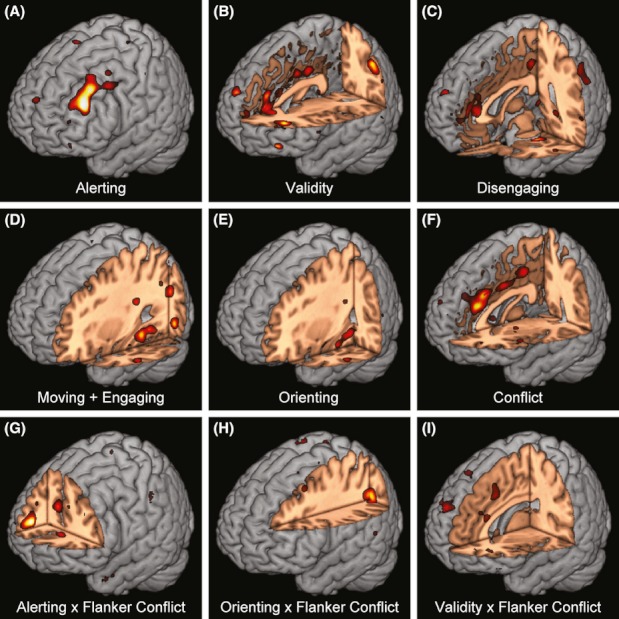
Differences (healthy controls [HC] greater than individuals with autism spectrum disorders [ASD]) in brain activation corresponding to the measures of network effects. The color was scaled from *t* >2.51 to 5 for these group difference maps.

Figure 3 and Table 2 show differences in brain activation between HC and ASD groups (HC > ASD) related to each of the three attentional processes; HC exhibited greater activation across all contrasts. For the alerting effect, the left MFG (Fig. 3A), caudate nucleus, and right MFG were significantly different. For the validity effect, mid/posterior cingulate cortex and pregenual ACC (Fig. 3B) in the fronto–parieto–cingulate network were significantly different. Further partition of the validity effect into its two subcomponents, disengaging and moving/engaging, showed that the left and right pregenual ACC (Fig. 3C), right supramarginal gyrus and inferior parietal lobule (IPL – a subdivision of TPJ), and angular gyrus were significantly different during disengaging, and that the fusiform gyrus (Fig. 3D), superior temporal gyrus, and anterior insular cortex were significantly different during moving/engaging. Orienting showed similar group differences (Fig. 3E) to the moving/engaging effect. The conflict effect showed focal differences in ACC activation (Fig. 3F).

**Table 2:** Greater network-related activation in HC compared with individuals with ASD

			MNI coordinates				
					
Region	L /R	BA	*x*	*y*	*z*	*Z*	*k*
Alerting^1^							
Mid-frontal gyrus	L	9	−38	36	28	4.63	650
Mid-frontal gyrus	L	46	−36	28	40	4.14	
Mid-frontal gyrus	L	9	−46	16	44	3.20	
Caudate nucleus	R		6	6	2	4.30	488
Caudate nucleus	L		−12	18	14	3.70	
Medial/orbital frontal gyrus	R	11	12	48	−12	3.98	182
Medial/orbital frontal gyrus	R	11	12	22	−8	3.34	
Medial/orbital frontal gyrus	R	11	18	34	−14	2.73	
Validity^2^							
Mid-frontal gyrus	L	9	−36	44	14	4.75	344
Mid-frontal gyrus	L	10	−24	46	4	3.02	
Inferior parietal lobule	L	40	−46	−52	48	3.74	188
Superior frontal gyrus	R	9	22	52	26	3.55	238
Mid-frontal gyrus	R	10	26	44	20	3.39	
Anterior cingulate cortex	L	24	0	36	26	3.52	627
Anterior cingulate cortex	R	24	4	34	12	3.49	
Anterior cingulate cortex	L	32	−8	36	22	3.33	
Inferior parietal lobule	R	40	48	−46	48	3.25	458
Supramarginal gyrus	R	40	54	−46	38	3.24	
Inferior parietal lobule	R	39	52	−54	40	3.02	
Mid-cingulate cortex	R	23	8	−16	38	3.24	261
Mid-cingulate cortex	L	23	0	−14	38	3.22	
Mid-cingulate cortex	R	24	4	−2	38	3.19	
Moving + Engaging^3^							
Fusiform gyrus	L	37	−34	−38	−10	3.92	509
Fusiform gyrus	L	19	−38	−70	−8	3.81	
Fusiform gyrus	L	37	−26	−40	−12	3.76	
Superior temporal gyrus	R	38	32	8	−30	3.48	167
Anterior insular cortex	R		32	16	−18	3.17	
Disengaging^1^							
Anterior cingulate cortex	L	24	−6	30	18	3.10	480
Anterior cingulate cortex	L	24	−2	34	10	3.05	
Anterior cingulate cortex	R	32	10	40	8	2.99	
Supramarginal gyrus	R	40	48	−40	36	2.84	301
Inferior parietal lobule	R	40	46	−46	44	2.80	
Angular gyrus	R	40	56	−50	30	2.73	
Orienting^1^							
Fusiform gyrus	L	37	−26	−24	−20	3.70	286
Fusiform gyrus	L	37	−34	−38	−10	3.63	
Anterior insular cortex	R		30	8	−14	3.47	213
Superior temporal gyrus	R		32	8	−28	3.28	
Flanker conflict^1^							
Anterior cingulate cortex	L	32	−2	34	24	3.91	1101
Anterior cingulate cortex	L	24	0	28	32	3.68	
Anterior cingulate cortex^4^	R	24	2	2	36	3.30	
Alerting by flank conflict							
Superior frontal gyrus	R	9	20	50	14	4.36	220
Orienting by flanker conflict							
Inferior parietal lobule	L	19	−32	−60	30	3.71	244
Mid-occipital gyrus	L	39	−38	−68	28	3.29	
Cerebellum (vermis)	L		−6	−74	−18	3.58	193
Validity by flanker conflict							
Anterior cingulate gyrus	R	32	4	14	46	2.92	157
Anterior cingulate gyrus	L	32	−6	16	40	2.82	

ASD, autism spectrum disorder; HC, healthy control; BA, Brodmann area; L/R, left/right; MNI, Montreal Neurological Institute.

1 There was no cluster showing significant greater activation for the contrast of ASD minus HC.

2 The contrast of HC minus ASD for validity showed cerebellum activation (*x* = −18, *y* = −56, *z* = −26, *Z* = 3.58, *k* = 209).

3 The contrast of HC minus ASD for Moving + Engaging showed left mid-frontal gyrus activation (Brodmann area 9, *x* = −38, *y* = 28, *z* = 38, *Z* = 3.95, *k* = 235).

4 Extends to the posterior cingulate cortex.

Interactions showed similar patterns to main effects. The alerting by flanker conflict effect was associated with greater activation in the right superior frontal gyrus (Fig. 3G); the orienting by flanker conflict effect was associated with greater activation in the IPS (Fig. 3H), mid-occipital gyrus, and cerebellar vermis; the validity by flanker conflict effect was associated with greater ACC activation (Fig. 3I).

### Further analysis of conflict processing and the executive control network

**Figure 4:**
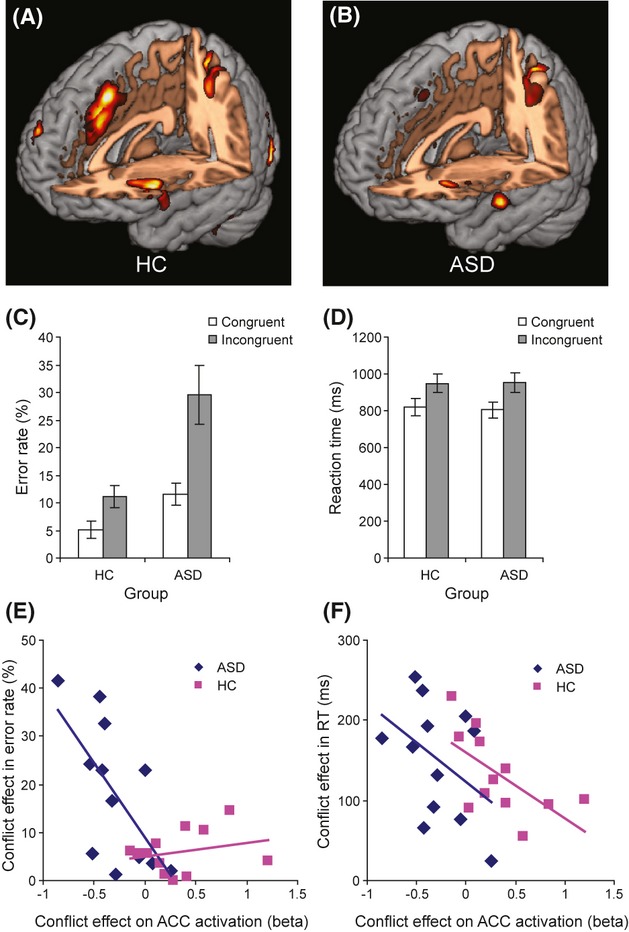
Brain activation associated with flanker conflict effect in healthy controls (HC) (A) and individuals with autism spectrum disorders (ASD) (B) during the attention network test. The color was scaled from *t* >2.51 to 5 for individual group maps. Behavioral performances measured by error rate (C) and reaction time (RT) (D) under congruent and incongruent conditions for the HC and ASD groups. Error bars represent the standard error under each condition; analyses of equality of the linear relationship between conflict effects in error rate and ACC activation (E), and between conflict effects in RT and ACC activation (F), in HC and ASD groups.

We hypothesized that executive control network abnormality in ASD was associated with deficits in the three domains of ASD. Therefore, we further examined patterns of group differences in conflict processing. Regions of the frontoparietal control network and the anterior insular cortex were activated in both groups (Fig. 4A and B, Tables 3 and 4). HC had greater activation than ASD only in the ACC (as in Fig. 3F and Table 2), with no significant activation in the ACC for conflict processing in the ASD group. There was also no cluster showing significantly greater activation for the contrast of ASD minus HC. In addition, the ACC cluster of group differences extended to the posterior cingulate cortex, which was due to greater deactivation in the ASD group. We also examined the possibility of a group (ASD, HC) by flanker congruency (congruent, incongruent) interaction by extracting perimeter estimates (β value) from the ACC. The HC group showed less deactivation for the incongruent condition than the congruent condition, resulting in a positive conflict effect. However, the ASD group showed greater activation for the congruent compared with the incongruent conditions, resulting in a negative (or lack of) conflict effect.

**Table 3:** Conflict-related activation in healthy controls

			MNI coordinates				
					
Region	L /R	BA	*x*	*y*	*z*	*Z*	*K*
Inferior parietal lobule^1^	R	7	26	−48	52	5.07	1679
Superior parietal lobule	R	7	22	−62	58	4.29	
Inferior parietal lobule	R	40	36	−40	52	4.15	
Inferior frontal/orbitofrontal gyrus^2^	R	47	50	20	−4	4.73	611
Anterior insular cortex	R		34	26	0	2.99	
Inferior frontal/orbitofrontal gyrus	R	47	36	24	−12	2.78	
Inferior occipital gyrus	L	19	−42	−68	−12	4.61	981
Cerebellum (Crus 1)	L		−36	−62	−28	3.75	
Inferior occipital gyrus	L		−44	−82	−4	3.73	
Superior occipital gyrus	R	19	36	−76	8	4.19	195
Mid-occipital gyrus	R	18	34	−84	6	3.65	
Inferior occipital gyrus	R	19	38	−84	−4	3.39	
Anterior insular cortex^2^	L		−34	18	−10	3.89	603
Anterior insular cortex	L		−42	16	−6	3.89	
Anterior insular cortex	L		−34	22	−2	3.22	
Anterior cingulate cortex	R	32	4	16	46	3.89	1084
Anterior cingulate cortex	R	24	4	22	34	3.63	
Supplementary motor area	L	6	−8	2	52	3.29	
Precentral gyrus	L	6	−30	−10	52	3.74	363
Precentral gyrus	L	6	−26	−6	46	3.56	
Precentral gyrus	L	6	−34	−8	42	2.94	
Superior frontal gyrus^3^	R	6	26	0	52	3.66	329
Precentral gyrus	R	6	44	0	44	2.99	
Precentral gyrus	R	6	40	−2	52	2.61	
Mid-occipital gyrus	R	19	30	−66	34	3.65	189
Mid-frontal gyrus	R	46	28	48	16	3.41	251
Mid-frontal gyrus	R	46	30	52	26	3.38	
Superior parietal lobule^1^	L	7	−26	−50	52	3.37	404
Precuneus	L	5	−10	−56	58	3.35	
Superior parietal lobule	L	7	−24	−44	46	3.16	
Inferior frontal gyrus	R	44	54	14	32	3.19	218
Inferior frontal gyrus	R	45	48	24	22	3.07	
Precentral gyrus	R	6	44	2	34	2.73	
Postcentral gyrus	L	2	−38	−34	42	3.10	189
Postcentral gyrus	L	2	−36	−38	58	2.92	

L/R, left/right; BA, Brodmann area; MNI, Montreal Neurological Institute.

1 Area along and near the intraparietal sulcus.

2 Frontoinsular cortex cluster.

3 Frontal eye fields.

**Table 4:** Conflict-related activation in individuals with ASD

			MNI coordinates				
					
Region	L /R	BA	*x*	*y*	*z*	*Z*	*K*
Anterior insular cortex^2^	R		32	16	4	4.59	807
Inferior frontal/orbitofrontal gyrus	R	47	42	22	−12	3.41	
Mid-occipital gyrus	R	19	34	−84	2	3.83	177
Inferior frontal gyrus	R	44	52	10	30	3.63	400
Inferior frontal gyrus	R	44	38	8	32	3.05	
Inferior frontal gyrus	R	44	46	26	30	2.95	
Mid-occipital gyrus	L	19	−24	−64	32	3.61	1063
Superior parietal lobule^1^	L	7	−28	−52	58	3.40	
Inferior parietal lobule	L	40	−32	−52	42	3.37	
Anterior insular cortex^2^	L		−32	26	2	3.37	187
Anterior insular cortex	L		−32	16	8	3.14	
Inferior parietal lobule^1^	R	40	32	−50	44	3.10	788
Inferior parietal lobule	R	19	30	−62	32	3.09	
Superior parietal lobule	R	7	30	−62	58	2.84	
Precentral gyrus	L	6	−44	0	26	3.06	145
Precentral gyrus	L	44	−50	6	32	2.80	

ASD, autism spectrum disorder; L/R, left/right; BA, Brodmann area; MNI, Montreal Neurological Institute.

1 Area along and near the intraparietal sulcus.

2 Frontoinsular cortex cluster.

Analysis of variance for the behavioral data was conducted with group (HC, ASD) as a between-subject factor and congruence (congruent, incongruent) as a within-subject factor. There was a significant main effect of conflict on error rate (*F*_(1, 22)_ = 29.63, *P* < 0.01); error rate under the incongruent condition was significantly higher than under the congruent condition. There was also a significant group difference on overall error rate (*F*_(1, 22)_ = 10.49, *P* < 0.01). In addition, the conflict by group interaction was significant (*F*_(1, 22)_ = 7.62, *P* = 0.01); the conflict effect was significantly greater in the ASD group than in the HC group. For RT, although the main conflict effect was significant (*F*_(1, 22)_ = 121.88, *P* < 0.01), the group difference was not significant (*F* < 1) and the conflict by group interaction was not significant (*F* < 1) (see Fig. 4C and D).

The conflict effect in error rate can be predicted by the conflict-related ACC activation (*r* = 0.56, *F*_(1, 22)_ = 9.81, *P* < 0.01). To examine whether the relation of conflict-related ACC activity and error rate between groups were parallel, the conflict effect in error rate was regressed on ACC activation, group, and ACC activation-by-group variables. The interaction term was significant (*t* = −3.16, *P* < 0.01), indicating that the slopes were not parallel. Further examination of the relation between conflict-related ACC activity and error rate by group showed a significant correlation in the ASD group (*r* = −0.66, *F*_(1, 10)_ = 7.80, *P* < 0.05), but not in the HC group (*r* = 0.26, *F* < 1). These results suggest that an increased cost of conflict (in error rate) is correlated with decreases in ACC activation in the ASD group, but no significant relation in the HC group (see Fig. 4E).

Similar to error rate, the conflict effect in RT can be predicted by conflict-related ACC activation (*r* = −0.46, *F*_(1, 22)_ = 6.04, *P* < 0.05) in both groups. More efficient conflict processing (less increase in RT under the incongruent condition compared with the congruent condition) was related to greater ACC activation. The interaction term in a model testing the parallelism of the two slopes with conflict-related ACC activation, group, and conflict-related ACC activation-by-group interaction as predictors showed that the interaction term was not significant (*t* = −0.23, *P* > 0.05). This indicates that the conflict-related ACC activation does not differentially predict the conflict effect in RT between groups (see Fig. 4F). ACC activity was related to the conflict effect measured by RT in both groups.

**Figure 5:**
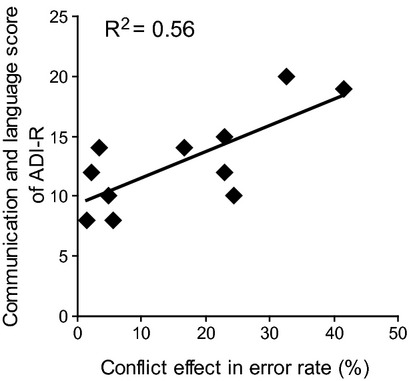
Symptom-executive control association. More symptoms of communication/language are related to greater cost on accuracy in conflict processing.

The relation between functional activation during the conflict processing of the ROI, which was identified by group difference, the behavioral effect of conflict, and ADI-R subscores in ASD group was also examined. Results indicate that the communication and language domain was significantly correlated with the efficiency (measured as accuracy) during conflict processing (Fig. 5). That is, domain symptoms in communication and language are related to less efficient conflict processing.

## Discussion

Our results indicate significant behavioral deficits of the alerting and executive attentional networks in ASD relative to HC, but not the orienting network or network interactions. Behavioral deficits were associated with abnormalities in the neural networks supporting attentional functions. Even in the absence of behavioral differences among the orienting network and network interactions, neural differences were present.

Individuals with ASD made more errors if there was no alerting cue preceding the target. This alerting deficit was associated with abnormal activation of MFG and caudate nucleus in ASD. The reduced activation of MFG and caudate nucleus may suggest a deficiency of using these brain areas for the alerting response to unanticipated targets in ASD. Deficits in these brain networks may underpin the abnormal alerting behavior identified in the present and previous studies (e.g., Pascualvaca et al. 1998). It is worth noting that unlike prior studies (Dawson et al. 1998; Landry and Bryson 2004; Teder-Salejarvi et al. 2005), we did not find significant group differences in behavioral effects of orienting.

For orienting, while behavior was similar between groups, differences in the neurophysiological data deserve further discussion. Greater activation for the validity effect (and subcomponents of disengaging and moving/engaging in key regions of the default-mode network (DMN) (mid/posterior cingulate cortex, and pregenual ACC, superior temporal gyrus, and angular gyrus) as well as in regions of the task-positive network (TPN) (anterior insular cortex, TPJ, IPL, and fusiform gyrus) for the HC > ASD contrast may indicate more task-related effort (decreased DMN, increased TPN) in the ASD group. This greater task-related effort could imply a form of compensation for behavioral performance in orienting. Inconsistencies in orienting deficits may be attributable to at least two major factors: (1) cerebellar and/or parietal abnormalities, not present in ASD patients in the present sample, are a likely contributor to orienting deficits (Townsend et al. 1996a); (2) recent evidence suggests that orienting deficits in ASD may be more related to social than nonsocial cues (Greene et al. 2011), a factor that could explain the lack of orienting deficits in this study (nonsocial cues were used), as well as inconsistencies in the literature.

Our results also show significant behavioral deficits of the executive control network in ASD relative to HC. Significant group differences in conflict processing of executive control were associated with, as hypothesized, abnormal ACC activation in ASD. However, unlike previous studies, we found an absence of ACC activation rather than hypoactivation. In addition, higher error rates were associated with the lack of activation in the ACC in ASD. That is, dysfunction of the ACC resulted in a higher error rate. Conflict-related ACC activation was negatively correlated with the conflict effect measured in RT, suggesting that ACC activation is related to efficiency of resolving conflict. Furthermore, increased number of symptoms in the domain of communication and language was related to less efficient conflict processing. Overall, these results indicate both behavioral and neural abnormalities in the executive control of attention in ASD and a direct association with symptom domains in ASD.

The significant ACC deficit during conflict processing may represent a fundamental deficit in ASD. This study shows abnormal (in fact, absent) ACC activation in ASD relative to HC in the anterior rostral cingulate zone (RCZa), a “cognitive” region of the ACC. Reduced metabolism (Haznedar et al. 1997) and reduced fractional anisotropy in white matter underlying the ACC (indicating abnormal microstructural integrity of the white matter) in ASD (Thakkar et al. 2008), and new evidence from our recent magnetic resonance spectroscopy study of the attentional networks in ASD showing lower glutamate/glutamine concentration in the right ACC (Bernardi et al. 2011), may explain this absence of ACC activation during conflict processing. Previous studies on ASD have also shown hypoactivation in the RCZa for conflict processing to response shifts (Shafritz et al. 2008), social–cognitive stimuli (Dichter and Belger 2007) and response inhibition (Kana et al. 2007), and reduced discrimination between errors and correct responses in a subregion defined as an affective division of the ACC (Bush et al. 2000). Higher error rates are typically related to greater ACC activation for conflict monitoring. While we found a negative correlation between ACC activation and error rates in the ASD group, there was no such correlation in the HC group. We speculate that decreased ACC activity is associated with low awareness (which is also associated with more errors), particularly in individuals with ASD.

The ACC, coupled with other brain areas such as the anterior insular cortex, plays a major role in executive control of attention (Bush et al. 2000; Posner and Fan 2008), response selection, preparation, execution (Frith et al. 1991), and emotion (Bush et al. 2000). Lack of control may lead to deficits in reciprocal social interaction, communication and language, and repetitive, stereotyped activity, as well as other behaviors commonly associated with autism. The current finding of an intact frontoparietal network in conflict processing in ASD distinguishes the ACC from the frontoparietal network, consistent with recent work by other groups (e.g., Dosenbach et al. 2008). It has been suggested that the ACC is involved in rapid information processing, whereas the frontoparietal network underpins more deliberate, adaptive control (Garavan et al. 2002; Kana et al. 2007; Dosenbach et al. 2008). Deficits in attentional domains may manifest when there is a requirement for rapid executive control during conditions involving high demands on information processing.

Although alterations in ACC activity are not specific to ASD, the heterogeneity of autistic symptoms may be related to ASD-specific abnormalities in structural and functional connectivity of the ACC with other brain structures and networks interacting with different cognitive domains. One recent study has shown that ASD is associated with deficits in the frontoparietal network, related to executive control (Solomon et al. 2009). However, current results indicate that the deficit is more localized; between-group differences in other regions such as the frontoparietal network and the anterior insular cortex were not significant. Further examination of the present attentional network deficits in ASD relative to other neurodevelopmental and psychiatric disorders will be necessary to test the specificity of the present patterns. Although deficits in the MFG and caudate are tentative, given few studies specifically examining these regions relating to alerting, the ACC abnormality may constitute a fundamental deficit which is related to other cognitive domains. Knowledge of deficits in alerting and executive control could be used to facilitate new adjunctive interventions for individuals with ASD, thus satisfying an important initiative to develop ASD-specific neurobehavioral domains.
